# Reporter gene imaging identifies intratumoral infection voids as a critical barrier to systemic oncolytic virus efficacy

**DOI:** 10.1038/mto.2014.5

**Published:** 2014-12-10

**Authors:** Amber Miller, Lukkana Suksanpaisan, Shruthi Naik, Rebecca Nace, Mark Federspiel, Kah Whye Peng, Stephen J Russell

**Affiliations:** 1 Mayo Graduate School, Mayo Clinic, Rochester, Minnesota, USA; 2 Department of Molecular Medicine, Mayo Clinic, Rochester, Minnesota, USA; 3 Imanis Life Sciences, Rochester, Minnesota, USA; 4 Department of Obstetrics and Gynecology, Mayo Clinic, Rochester, Minnesota, USA; 5 Division of Hematology, Department of Medicine, Mayo Clinic, Rochester, Minnesota, USA

## Abstract

Systemically administered oncolytic viruses have the ability to cause tumor destruction through the expansion and coalescence of intratumoral infectious centers. Efficacy is therefore dependent upon both the density and intratumoral distribution of virus-infected cells achieved after initial virus infusion, and delivery methods are being developed to enhance these critical parameters. However, the three-dimensional (3D) mapping of intratumoral infectious centers is difficult using conventional immunohistochemical methodology, requiring painstaking 3D reconstruction of numerous sequential stained tumor sections, with no ability to study the temporal evolution of spreading infection in a single animal. We therefore developed a system using very high-resolution noninvasive *in vivo* micro single-photon emitted computed tomography/computed tomography (microSPECT/CT) imaging to determine the intratumoral distribution of thyroid radiotracers in tumors infected with an oncolytic virus encoding the thyroidal sodium–iodide symporter (NIS). This imaging system was used for longitudinal analysis of the density, distribution, and evolution of intratumoral infectious centers after systemic administration of oncolytic vesicular stomatitis virus in tumor-bearing mice and revealed heterogeneous delivery of virus particles both within and between tumors in animals receiving identical therapy. This study provides compelling validation of high resolution *in vivo* reporter gene mapping as a convenient method for serial monitoring of intratumoral virus spread that will be necessary to address critical barriers to systemic oncolytic virus efficacy such as intratumoral delivery.

## Introduction

Oncolytic viruses, such as vesicular stomatitis virus (VSV), are novel, experimental cancer therapeutics that selectively extravasate into tumors to infect and kill cancer cells.^1^ Systemic, intravenous administration allows the viruses to reach distant or multiple metastases, and efficacy is dependent on sufficient infectious virus particles reaching sites of tumor growth. It has been predicted mathematically and confirmed experimentally that there is a virus dose threshold necessary to establish infection in the tumor.^2,3^ Reduction in virus bioavailability limits the amount of virus that reaches the tumor to initiate infection. Virus bioavailability is rapidly reduced by antibody and complement neutralization in the blood, sequestration in the mononuclear phagocytic system of the liver and spleen, off-target extravasation at sites other than the targeted tumors and failure of extravasation in the tumors.^1^ Approaches are being developed to limit the influence of these barriers, thereby improving virus delivery to the tumor microvessels,^1,4,5^ but antitumor efficacy ultimately depends on its homogeneous dissemination throughout the tumor parenchyma. In the systemic oncolytic virotherapy trials reported to date, detectable levels of intratumoral virus delivery have been highly variable and only a small number of complete tumor responses have been documented.^6,7^ We are therefore working to optimize the potency of systemic oncolytic virotherapy by developing new protocols for virus infusion that will increase the efficiency and uniformity of intratumoral virus delivery.

To evaluate the relative merits of different virus infusion protocols, it is necessary to determine how the virus distributes throughout tumors in time and space under different experimental conditions. The traditional approach to this question is to harvest tumors at multiple timepoints after virus administration and section and stain them with antiviral antibodies. This approach, however, gives only two-dimensional (2D) data, and while painstaking three-dimensional (3D) reconstruction of the data from multiple serial sections is theoretically possible, the approach is too time consuming to be of real practical value. An additional serious limitation of this traditional approach is that the tumor-bearing animal must be killed at the time of tumor harvest making it impossible to follow the evolution of oncolytic infection in a single animal over time.

Recombinant viruses engineered to encode reporter proteins whose expression can be detected by noninvasive radioisotopic imaging methods have been used extensively to enable the noninvasive quantitative monitoring of viral infection.^8^ One such reporter gene currently used in preclinical and clinical applications is the sodium–iodide symporter (NIS) gene which codes for a protein that concentrates iodide and other anionic radiotracers in virus-infected cells of live animals that is detected by nuclear imaging techniques including positron emission tomography imaging and single-photon emitted computed tomography (SPECT) imaging.^9^ Indeed, the NIS reporter gene has been used extensively to track the intratumoral propagation of recombinant oncolytic viruses such as measles, vesicular stomatitis, vaccinia, herpes simplex, and adenoviruses, although the resolution of the imaging data reported in these studies was relatively limited.^10–19^ Resolution is the ability to distinguish between two closely spaced objects on an image. Since positrons travels a short distance before annihilation, the theoretical maximum resolution of positron emission tomography is inferior to SPECT, a fact that is (at last) reflected in the performance of the newest microSPECT instruments. However, resolution comes at the expense of sensitivity in SPECT imaging; so, there is an important tradeoff between these two critical parameters. Advances in imaging technology now allow for not only considerably higher sensitivity of γ-ray detection at the body surface but also for much higher resolution of adjacent foci of NIS radiotracer uptake within a given tumor.

We therefore decided to assess the ability of high-resolution micro single-photon emitted computed tomography/computed tomography (microSPECT/CT), using our recently acquired USPECT-II/CT imaging instrument (MILabs) with theoretical resolution <0.35 mm compared to the resolution of our Siemens Inveon positron emission tomography/CT with theoretical resolution <1.4 mm, to track the evolution of intratumoral infectious centers after systemic administration of a NIS-expressing oncolytic VSV (VSV-mIFNβ-NIS) in tumor-bearing mice.^20,21^ Here, we report the initial findings of these studies, which provide a strong validation for the use of high-resolution *in vivo* reporter gene mapping to serially monitor the intratumoral spread of an oncolytic virus infection. The data also highlight the problem of heterogeneous delivery of virus particles both within and between tumors in animals receiving identical therapy.

## Results

### High-resolution microSPECT/CT imaging of VSV-mIFNβ-NIS-treated mice shows multiple, heterogeneously distributed intratumoral foci of radiotracer concentration

**Figure 1:**
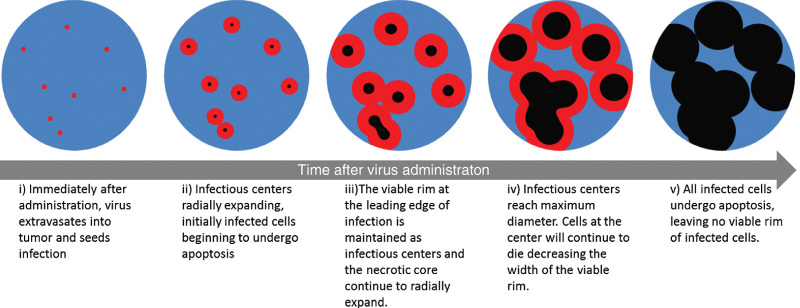
Diagrammatic illustration of the progression in intratumoral oncolytic virus infection. The stages of infection depicted here correlate with the changes in radiotracer uptake concentration that is detected with autoradiography and microSPECT/computed tomography (CT) imaging analysis. (i) Multiple infectious centers (red) are seeded throughout the tumor (blue). (ii) Each infectious center expands radially as the infection spreads outward. After ~20 hours, infected cells at the core of the infectious center begin to die (black) leaving a rim of viable infected cells that correlate with maximum radiotracer uptake. (iii) As infection continues to spread, the infectious centers continue to enlarge but the rim of viable cells is maintained. Centers of infection coalesce to form large regions of infection. (iv) Infection reaches its maximum spread and (v) all infected cells die, corresponding to loss of radiotracer uptake.

We previously proposed and experimentally validated a mathematical model describing the radial expansion and conflation of independent spherical intratumoral infectious centers in mouse plasmacytomas treated intravenously with an oncolytic VSV encoding the NIS reporter gene.^2^ The model was based on immunohistochemical analysis showing that virus seeds intratumoral infection that spreads outward to neighboring cells with infected cells remaining viable before becoming apoptotic. Functional NIS expression has previously been shown to correlate with these stages of infection as infected cells express the NIS transgene for ~20 hours before becoming apoptotic and losing their ability to concentrate radioiodine. Autoradiography of explanted tumors from multiple timepoints after infection showed that radiotracer accumulation was maximal at the periphery of expanding infectious centers, corresponding to the leading edge of the advancing infection where the tumor cells had only recently been infected and were therefore still viable (Supplementary Figure S1).^19^ This validates the ability to detect changes in infection over time using detection of radiotracer concentration, and these stages of infection are diagrammatically illustrated in Figure 1. However, because 3D imaging is better suited to depict virus–tumor interactions than immunohistochemical or autoradiographic analysis, our aim was to validate the use of high-resolution, noninvasive imaging to depict all aspects of intratumoral infection.

**Figure 2:**
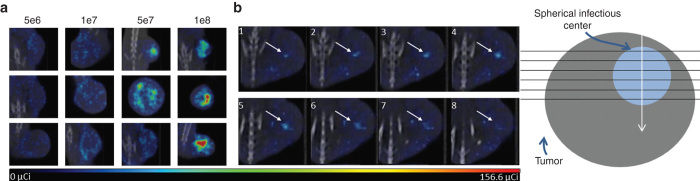
High-resolution microSPECT/computed tomography (CT) imaging is able to distinguish individual centers of radiotracer uptake. (**a**) Single planes from microSPECT/CT imaging of three different tumor-bearing mice 24 hours after intratumoral vesicular stomatitis virus (VSV)-mIFNβ-NIS infection at doses 5 × 10^6^ through 1 × 10^8^ TCID_50_. The microSPECT/CT imaging is able to discern individual foci of radiotracer uptake. The distribution and density of the individual foci is dependent on virus dose although variability across mice given the same treatment is seen. (**b**) Serial planes along a single axis through the tumor of an MPC-11 tumor-bearing immunocompetent BALB/c mouse 24 hours after 5 × 10^6^ TCID_50_ VSV-mIFNβ-NIS shows increasing and decreasing diameter and intensity of a single radiotracer uptake center indicating approximately spherical geometry. Diagram shows the collection and orientation of serial planes.

To determine whether high-resolution microSPECT/CT imaging could be used to detect and resolve multiple individual intratumoral infectious foci, immunocompetent BALB/c mice bearing subcutaneous MPC-11 plasmacytomas (5 mm approximate diameter) were given a single intravenous dose of VSV-mIFNβ-NIS (5 × 10^6^ to 1 × 10^8^ TCID_50_) and imaged 24 hours later. The microSPECT/CT data were collected over a period of 25 minutes, starting 1 hour after intraperitoneal administration of 500 µCi ^99m^Tc as the pertechnetate anion (^99m^TcO4^−^). The results of these imaging studies show that microSPECT/CT imaging is able to distinguish multiple independent foci of radiotracer uptake by VSV-mIFNβ-NIS-infected cells within a single tumor (Figure 2 and Supplementary Video S1 for 3D rotation). The shapes of these infectious centers can be appreciated by comparison of serial sections along one axis of the tumor through a single infectious center, which show a changing diameter and intensity of radioactive uptake most consistent with a spherical geometry (Figure 2b). In addition, it can be appreciated that there is significant heterogeneity in the diameters of the discernible infectious centers at this 24-hour timepoint. The shapes and sizes of the intratumoral infectious centers can be more readily appreciated in a rotating 3D rendering of the data from this experiment, shown in Supplementary Video S1.

To further determine whether noninvasive NIS imaging could be used to evaluate the density and heterogeneity of intratumoral virus distribution after intravenous VSV-mIFNβ-NIS administration, imaging studies performed on tumor-bearing mice imaged 24 hours after intravenous administration of varying doses of VSV-mIFNβ-NIS were compared. Figure 2 shows the distribution of intratumoral foci of radiotracer accumulation in the tumors of mice treated at doses of 5 × 10^6^, 1 × 10^7^, 5 × 10^7^, and 1 × 10^8^ TCID_50_ VSV-mIFNβ-NIS 24 hours after virus administration. The density of intratumoral foci of infection is clearly related to the dose of virus administered, but for a given dose of virus, there is considerable variability both in the density and in the distribution of intratumoral foci. The heterogeneity observed in infectious center diameters and in distribution patterns results in intratumoral infectious voids of viable tumor tissue as detected by positive nuclear Hoechst staining (Supplementary Figure S2). The shape and significance of these voids, however, cannot be fully appreciated by conventional immunohistochemical approaches.

### Serial microSPECT/CT imaging studies to monitor the evolution of an intratumoral VSV-mIFNβ-NIS infection

**Figure 3:**
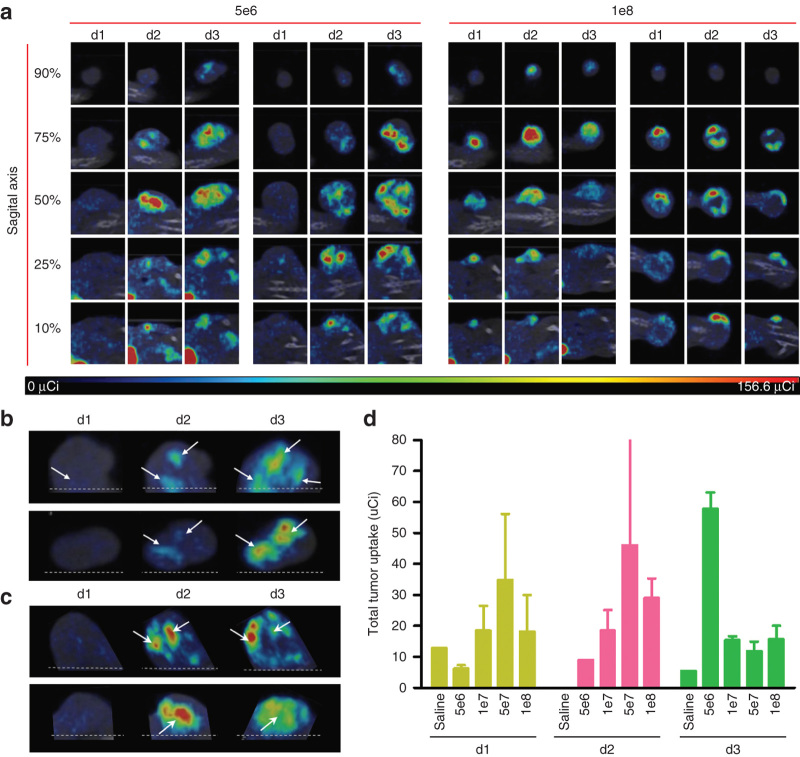
Patterns of intratumoral infection spread in live animals. (**a**) Live microSPECT/computed tomography (CT) imaging of intratumoral vesicular stomatitis virus (VSV)-mIFNβ-NIS over time in two separate animals at two different intravenous virus doses (5 × 10^6^ and 1 × 10^8^ TCID_50_). The sagittal planes have been selected from each tumor as depicted in Supplementary Figure S3 and are shown at each of three timepoints (24, 48, and 72 hours). (**b**) Selected planar images of microSPECT/CT imaging to show sequential expansion and conflation of infectious centers over time. (**c**) Selected planar images of microSPECT/CT imaging to illustrate loss of signal intensity from dying infectious centers. (**d**) Average tumor dosimetry as measured by total tumor radiotracer uptake determined days 1, 2, and 3 post virus administration for animals given intravenous virus at doses ranging from 5e6 to 1e8 TCID50 compared to average uptake in control mice given saline.

To determine whether high-resolution microSPECT/CT imaging could be used to follow the evolution of an intratumoral VSV-mIFNβ-NIS infection (*i.e.*, the expansion and conflation of intratumoral infectious centers), VSV-mIFNβ-NIS was again administered to immunocompetent BALB/c mice bearing subcutaneous MPC-11 plasmacytomas and microSPECT/CT images were collected from each mouse at multiple timepoints thereafter (24, 48, and 72 hours), always over a period of 25 minutes starting 1 hour after intraperitoneal administration of 500 µCi ^99m^TcO_4_^−^. The results of these imaging studies for two mice administered low (5 × 10^6^ TCID_50_) and high (1 × 10^8^ TCID_50_) doses of VSV-mIFNβ-NIS are shown in Figure 3 with 3D rotations in Supplementary Video S2. From these serial images, it is clear that the uptake and intratumoral distribution of the radioactive pertechnetate does change substantially as the infection evolves. At the earliest timepoint, 24 hours after virus administration, many of the infectious centers that later appear are not yet visible, and those that can be discerned are only faintly visualized, presumably because of their relatively small size, hence low uptake of pertechnetate at that early stage. Comparing the 24, 48, and 72-hour images from individual tumors, different patterns of signal evolution are observed. For the majority of foci examined, the size and intensity of the signal increased progressively through the 24, 48, and 72-hour timepoints (Figure 3b), indicating either continued radial expansion of the infectious centers and/or their coalescence with each other to form larger regions of infection. However, further analysis of the images reveals that not infrequently, the signal intensity for a given focus will increase from the 24 to the 48-hour timepoint, and then diminish by the 72-hour timepoint (Figure 3c). This is consistent with cellular apoptosis and loss of functional NIS expression. Again, analysis of these serial images from individual tumors highlights the variable distribution and density of infectious centers and the presence of variably sized regions within each tumor that appear to be void of infection.

Tumor dosimetry was completed to compare virus dose with radiotracer uptake over time (Figure 3d). This analysis confirmed that animals given low doses of virus had increasing radiotracer uptake over the 72-hour period while animals given higher doses of virus achieved maximum radiotracer uptake earlier and had decreased uptake intensity by 72 hours. This is consistent with the dose-dependent infection density and more rapid transition through the stages of infection at higher starting doses. Since cell death occurs after infection, it is assumed that tumors with an initially greater infection burden (greater uptake) also have a greater amount of cell death detectable by loss of uptake at earlier timepoints. This analysis supports the hypothesis that there is a relationship between virus dose and time of maximal uptake intensity. Mice given the lowest dose of virus, 5e6 TCID_50_, reached maximum tumor uptake at 72 hours. Mice given 5e7 TCID_50_ of virus reached maximum uptake at 48 hours. The trend of the data follows that mice given the intermediate virus dose, 1e7 TCID_50_, may have reached maximum uptake at sometime between the 48- and 72-hour imaging times, while mice given the highest dose, 1e8 TCID_50_, may have reached maximum uptake at sometime between the 24- and 48-hour imaging times. Additional timepoint analysis will be necessary to confirm this hypothesis.

### Space-filling models accurately reflect the distribution of intratumoral infection

**Figure 4:**
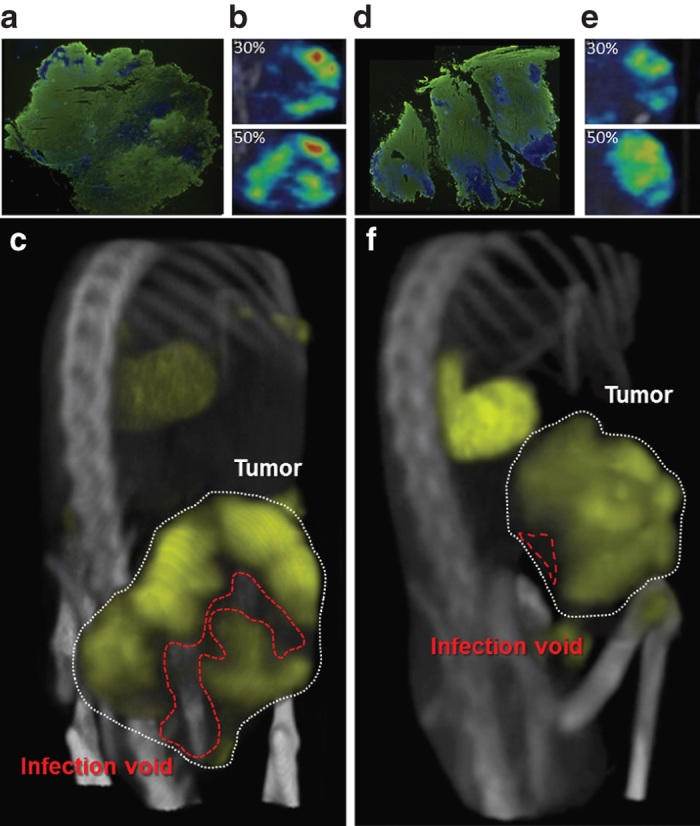
Space filling models show infection voids. The need for three-dimensional (3D) imaging is made clear when comparing tumors from multiple animals. (**a/d**) Immunohistochemical staining of tumors shows extensive vesicular stomatitis virus (VSV) infection (anti-VSV, green) throughout the tumor (Hoechst, blue). (**b/e**) Singular microSPECT/computed tomography (CT) planes taken at the same relative position within the same tumors as those stained in **a/d** show similar distribution relative to each other (top) but different planes through the same tumors at another location show different distributions. (**c/f**) 3D space filling allows differences in distribution to be clearly appreciated where tumor on top has decreased voids compared to tumor on bottom.

Spatial comparisons are achieved through 3D space-filling models that combine all planar microSPECT/CT images into a comprehensive volume rendering of intratumoral infection distribution. Space-filling models are necessary to gain spatial context and to visualize distributive relationships between infectious centers (Figure 4, Supplementary Video S3). The importance of this technology is clear when comparing conclusions made from singular immunohistochemical stained tumor sections or planar microSPECT/CT images with the space-filling models of the same animals. While 2D slices do not provide contextual information and may misrepresent the state of virus infection through the entire tumor, 3D space filling models clearly demonstrate infection throughout the entire tumor. For example, comparison of immunohistochemical staining of two different tumors after identical treatment with VSV-mIFNβ-NIS is deceiving as the sections from both tumors appear to show the same degree of infection (Figure 4a,d). Similarly, single SPECT/CT planes from each tumor show similar degrees of infection via radioactive uptake intensity (Figure 4b,e). However, comparison of additional planes shows discrepancies in intratumoral infection distribution that are only fully appreciated in space filling models (Figure 4c,f).

## Discussion

Infection voids are a serious problem both in the ability to analyze intratumoral infection dynamics and in the ability to adequately treat tumors with systemic oncolytic virotherapy. Infection voids are a direct result of the heterogeneity of intratumoral infection distribution, and the nonuniformity of delivery is confirmed here to be a common problem within and between tumors in animals receiving identical therapy. The resultant volumes of tissue void of infection contribute to lack of disease clearance and tumor regression. Therefore, understanding and overcoming the causes of variability in delivery undermines the ability to standardize and optimize therapies. In order to develop novel delivery techniques, imaging modalities that adequately portray intratumoral infection distribution are necessary for comparative analysis.

While *ex vivo* analysis of tumor sections can provide detailed depictions of virus–tumor interactions at the molecular level, descriptions of the distribution and spread of infection in individual tumors based on immunohistochemical staining of tumor sections cannot be obtained because this imaging modality is limited to single observations in time and space. Conventional immunohistochemical staining to observe 3D intratumoral virus delivery would require processing, staining, analysis, and painstaking 3D reconstruction of numerous sequential tumor sections. Our group has previously published on the correlation between the tomographic NIS imaging and parallel immunohistochemical analysis of tumors explanted immediately following noninvasive imaging.^20^ The methods used for this analysis are strikingly similar to those that would be used for a complete 3D reconstruction of conventional immunohistochemical staining. This is a very impractical method that still lacks the ability to study the temporal evolution of the oncolytic infection in a single animal that would not change the conclusions regarding intratumoral infection made here.

Due to the inability to portray the spatio-temporal context of intratumoral infection, conclusions regarding the spread of infection using *ex vivo* tissue analysis rely on information gathered from multiple tumors from multiple time points. This type of analysis does not allow for individual infections to be followed over time and merely allows for generalized observations of how viral infection progresses. *Ex vivo* analysis was used to develop our mathematical model predicting the probability of tumor survival after systemic oncolytic virotherapy, but assumptions regarding infection distribution and spread had to be made.^2^ The assumptions made for modeling purposes were that infectious centers are established after administration with no secondary viremia, they are randomly distributed throughout the tumor, they are spherical, they expand radially, and they coalesce with adjacent centers. The 3D imaging described here provides important validation of the latter assumptions by distinguishing separate infectious centers at early timepoints and allowing the same infectious centers to be followed through time to show radial expansion and conflation. Secondary viremia, or virus entering the blood stream to initiate additional infection is not likely to occur in this model during the timeframe in which imaging was performed and would not affect the outcome of the current imaging. Importantly, 3D imaging and space filling models presented here show that the distribution of infectious centers deviates from the uniform random distribution previously assumed from *ex vivo* analysis of tumor sections and will be a useful adjunct to future studies designed to address this problem.

This NIS imaging technology will be beneficial to compare changes in intratumoral infection distribution and overall tumor infection burden as a result of different infusion protocols. However, this technology has not yet been used to differentiate between infection resulting from sequential oncolytic virus administration. For differentiation between infectious centers from multiple doses, noninvasive reporter genes with specificity for different radioisotopes will be necessary. The potential to use SPECT imaging to detect different radiotracers, dual-tracer imaging, is being developed and could be used in the future with similar analysis techniques.^22^ This could allow for detection of different reporter gene combinations such as oncolytic virus expressing NIS and the herpes virus type 1 thymidine kinase (HSV1-TK) using both NIS-specific and HSV1-TK-specific radiotracers.^23,24^

The use of noninvasive imaging offers the benefits of spatial and temporal observation of intratumoral infection. This imaging technique is however limited in the ability to portray cellular states independent of infection status. The MPC-11tumor model used here may be a special case for observing intratumoral spread of oncolytic VSV leading to wholesale tumor destruction. In other tumor models, immune-mediated cell death or vascular shutdown resulting in bystander killing are additional sources of tumor cell death subsequent to oncolytic virus administration.^25,26^ The use of noninvasive imaging to observe intratumoral infection is unable to portray cell death not associated with direct virus infection. Therefore, immunohistochemical analysis remains a valuable tool to investigate roles of immune-mediated and bystander cell killing.

The heterogeneity of infectious center distribution throughout a tumor as well as the variability of distribution across multiple tumors requires the use of 3D, live-animal imaging beyond standard immunohistochemical techniques in order to analyze spatial or temporal virus–tumor interactions. The work presented here validates the use of noninvasive microSPECT/CT imaging to monitor VSV-mIFNβ-NIS infection distribution and evolution through time and space in tumor-bearing animals. The use of noninvasive imaging to track intratumoral infection is a convenient and accurate method of collecting anatomic and functional data of analytical value for optimizing and standardizing systemic oncolytic virotherapy. Importantly, the heterogeneity of oncolytic virus infection within a tumor hinders the comparison of infection distribution across multiple tumors and ultimately confirms the presence of tumor infection voids as a detriment to therapeutic efficacy. The heterogeneity of infection distribution identified here is a serious issue in the field of oncolytics. In order to advance oncolytic therapies, it will be necessary to optimize the predictability and homogeneity of virus delivery throughout every tumor, a pursuit in which 3D noninvasive imaging will be invaluable.

## Materials and Methods

### Cells

All cells were cultured at 37 °C in 5% CO_2_ atmosphere. MPC-11 murine myeloma cells and BHK-21 cells were obtained from and American Type Culture Collection (Manassas, VA) and cultured in Dulbecco’s modified Eagles medium (DMEM; Mediatech, Herndon, VA) supplemented with 10% FBS. All media contained penicillin (100 U/ml) and streptomycin (100 mg/ml) antibiotics. MPC-11 cells are syngeneic to the immunocompetent BALB/c mouse strain and successful tumor growth confirms cell line identity. These cell lines were not otherwise authenticated.

### Viruses

VSV-mIFNβ-hNIS^19^ was amplified in BHK-21 cells as previously described.^19^ Viral titer was quantified by measuring 50% tissue culture infective dose (TCID_50_) on BHK-21 cells calculated using the Spearman–Karber equation as described previously.^27^ Virus was stored at −80 °C until use.

### *In vitro* animal studies

Animals were maintained and cared for in accordance with Mayo Clinic Institutional Animal Care and Use Committee (A57712 and A23810). MPC-11 syngeneic murine myeloma tumors were established in mice by subcutaneously implanting washed tumor cells (5 × 10^6^ cells in PBS, total volume 100 µl) into the right hind flank of 5-to-6-week-old female immunocompetent BALB/c (Harlan, Indianapolis, IN) mice. Tumor burden was measured by serial caliper measurements. When the tumors reached 0.5 cm in diameter, a single dose of recombinant VSV in saline (total volume of 100 µl, ≤ 10^8^ TCID_50_/mouse) was administered intravenously via the tail vein.

### SPECT/CT imaging

A high-resolution microSPECT/CT system (U-SPECT-II, MI Labs, Utrecht, The Netherlands) was used for coregistered microSPECT/CT imaging 24–72 hours postvirus administration. A dose of 0.5mCi ^99m^TcO_4_^−^ was administered IP to animals 1 hour prior to imaging and then imaged for 25 minutes (5 minutes CT and 20 minutes SPECT). During imaging, animals were maintained under general anesthesia with isoflurane in O_2_ supplied from a veterinary vaporizer and delivered through mouse-specific nose cones. The resulting Neuroimaging Informatics Technology Initiative (NIFTI) image files were analyzed using PMOD 3.5 software (PMOD Technologies, Zurich, Switzerland). Data analysis, region of interest quantitation and video files were generated by Imanis Life Sciences (Rochester, MN).
