# Supplementary figures and images for: Reporter gene imaging identifies intratumoral infection voids as a critical barrier to systemic oncolytic virus efficacy
Source: Mol Ther Oncolytics. 2014 Dec 10;1:14005–. doi: 10.1038/mto.2014.5 (PMC4782940; doi:10.1038/mto.2014.5)

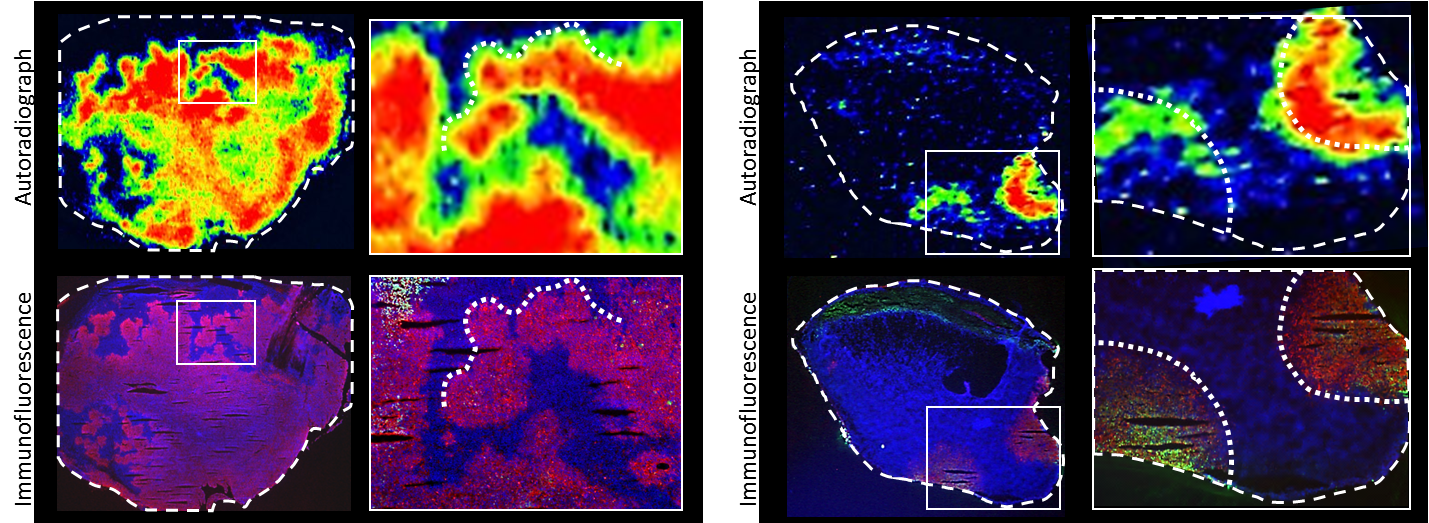

Supplement: Supplementary Figure S1 [file mto20145-s1.tiff]

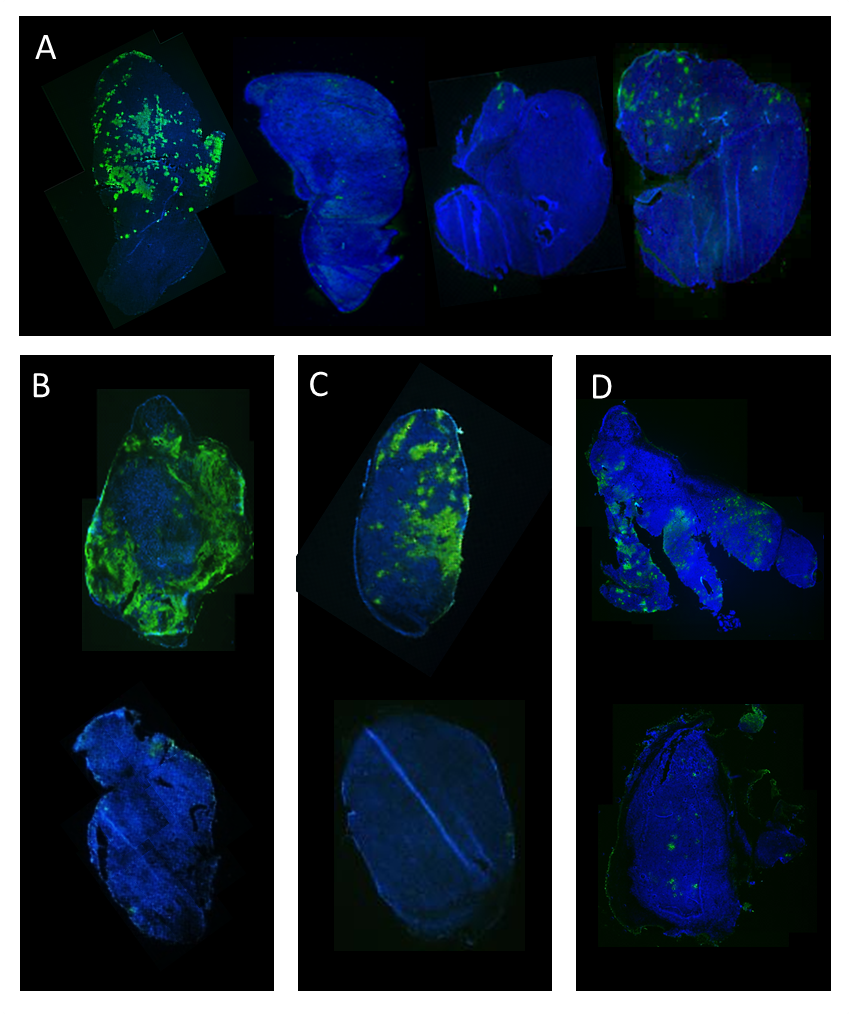

Supplement: Supplementary Figure S2 [file mto20145-s2.tiff]

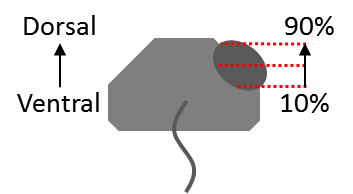

Supplement: Supplementary Figure S3 [file mto20145-s3.tiff]
